# Human induced pluripotent stem cells (hiPSCs) derived cells reflect tissue specificity found in patients with Leigh syndrome French Canadian variant (LSFC)

**DOI:** 10.3389/fgene.2024.1375467

**Published:** 2024-04-19

**Authors:** Roselle Gélinas, Chloé Lévesque, Julie Thompson Legault, Marie-Eve Rivard, Louis Villeneuve, Catherine Laprise, John D. Rioux

**Affiliations:** ^1^ Montreal Heart Institute, Montreal, QC, Canada; ^2^ Université du Québec à Chicoutimi, Saguenay, QC, Canada; ^3^ Université de Montréal, Montreal, QC, Canada

**Keywords:** induced pluripotent stem cells, three germ layers, hepatocyte-like cells, cardiomyocyte cells, disease modeling, Leigh syndrome French Canadian type

## Abstract

Leigh syndrome French Canadian type (LSFC) is a recessive neurodegenerative disease characterized by tissue-specific deficiency in cytochrome c oxidase (COX), the fourth complex in the oxidative phosphorylation system. LSFC is caused by mutations in the leucine rich pentatricopeptide repeat containing gene (*LRPPRC*). Most LSFC patients in Quebec are homozygous for an A354V substitution that causes a decrease in the expression of the LRPPRC protein. While LRPPRC is ubiquitously expressed and is involved in multiple cellular functions, tissue-specific expression of LRPPRC and COX activity is correlated with clinical features. In this proof-of-principle study, we developed human induced pluripotent stem cell (hiPSC)-based models from fibroblasts taken from a patient with LSFC, homozygous for the LRPPRC*354V allele, and from a control, homozygous for the LRPPRC*A354 allele. Specifically, for both of these fibroblast lines we generated hiPSC, hiPSC-derived cardiomyocytes (hiPSC-CMs) and hepatocyte-like cell (hiPSC-HLCs) lines, as well as the three germ layers. We observed that LRPPRC protein expression is reduced in all cell lines/layers derived from LSFC patient compared to control cells, with a reduction ranging from ∼70% in hiPSC-CMs to undetectable levels in hiPSC-HLC, reflecting tissue heterogeneity observed in patient tissues. We next performed exploratory analyses of these cell lines and observed that COX protein expression was reduced in all cell lines derived from LSFC patient compared to control cells. We also observed that mutant LRPPRC was associated with altered expression of key markers of endoplasmic reticulum stress response in hiPSC-HLCs but not in other cell types that were tested. While this demonstrates feasibility of the approach to experimentally study genotype-based differences that have tissue-specific impacts, this study will need to be extended to a larger number of patients and controls to not only validate the current observations but also to delve more deeply in the pathogenic mechanisms of LSFC.

## 1 Introduction

Leigh syndrome French Canadian (LSFC; OMIM#220111) is a recessive neurodegenerative infantile disease, lethal in most cases within the first 2 years of life, with high incidence in the Saguenay-Lac-St-Jean region of Quebec, (∼1/2,000 births; carrier rate 1/23) (Merante et al., 1993; Morin et al., 1993). LSFC is caused by mutations in the nuclear-encoded gene leucine rich pentatricopeptide repeat containing protein (*LRPPRC*) (Mootha et al., 2003). Most LSFC patients in Quebec are homozygous for an A354V substitution, except for one compound heterozygous patient that also carries a truncation variant in exon 35 that results in a premature stop at amino acid 1,277. Other variants in *LRPPRC* have been identified in unrelated families in Europe and China (Olahova et al., 2015; Han et al., 2017; Piro et al., 2020). Although associated with a variable phenotypic severity, at the molecular level, all patients exhibit a decrease in the steady-state level of the LRPPRC protein (Mootha et al., 2003; Xu et al., 2004).

LRPPRC is a ubiquitously-expressed mitochondrial protein that plays various biological roles via direct and indirect protein-protein interactions, including in energy metabolism and maturation of nuclear export of mRNA (Cui et al., 2019). LSFC is characterized by tissue-specific deficiency in cytochrome c oxidase (COX), the fourth complex in the oxidative phosphorylation (OXPHOS) system. COX activity is estimated at 10%–20% in brain and liver, 50% in skin fibroblasts and skeletal muscle, while close to normal in kidney and heart (Merante et al., 1993). Biomolecular analyses conducted in post-mortem tissues from LSFC patients also showed that loss of LRPPRC and its interacting protein SLIRP results in tissue-specific OXPHOS deficiency (Sasarman et al., 2015). Tissue-specific differences in the levels of mutant LRPPRC protein could thus partially explain the tissue heterogeneity.

To better understand the tissue-dependent impact of the *LRPPRC* mutation, additional model systems are needed. Not surprisingly germline knockout (KO) and knock in (KI) mouse models for *LRPPRC* are non-viable, with tissue-specific conditional KO are very challenging (e.g., neuron-specific model survives only a few days; hepatocyte-specific model is unstable over time as there is presence of residual amounts of LRPPRC which is likely attributable to liver regeneration). We were therefore interested in determining whether patient-derived hiPSC-based models (Takahashi and Yamanaka, 2006) could provide an opportunity to not only study pathogenic mechanisms but also the mechanisms by which certain organs are spared. Furthermore, considering that the mitochondrial complex assembly machinery is specific to humans, hiPSC-based models offer unique systems to study such diseases (for review (Inak et al., 2017)).

In this proof-of-principle study, hiPSCs were successfully reprogrammed from fibroblasts from one LSFC patient homozygous for LRPPRC*354V and one control. We used these cells to specifically assess the impact of the LRPPRC*354V mutation in (i) hiPSCs, (ii) cells from the three germ layers, namely, definitive endoderm, ectoderm and mesoderm, (iii) hiPSC-derived cardiomyocytes (hiPSC-CMs) and (iv) hiPSC-derived hepatocyte-like cells (hiPSC-HLCs). We observed tissue specific differences in LRPPRC expression in lines derived from the LSFC patient that were consistent with that observed in patient tissues. While these observations need to be extended to additional patients and control individuals, we propose that genotype-specific hiPSC-based models have the potential to help address the molecular basis of tissue specificity in pathophysiology of LSFC.

## 2 Material and methods

### 2.1 Antibodies and primers

Antibodies and primer sets were listed in Supplementary Tables S1, 2, respectively.

### 2.2 Cell culture of skin fibroblasts and fibroblast-derived hiPSCs

The study protocol was approved by the Research Ethics Committees of the CIUSSS du Saguenay–Lac-Saint-Jean and of the Montreal Heart Institute. Immortalized human skin fibroblast lines from one female LFSC patient and one female non-carrier control subject, aged 8 and 5 years respectively, were obtained from the LSFC Consortium Biobank (C. Laprise’s Laboratory at Université du Québec, Saguenay, QC, Canada). The skin biopsies had been previously obtained from subjects after receiving written informed consent from their legal guardian(s). Fibroblasts were immortalized as described in (Yao and Shoubridge, 1999) and maintained in DMEM (Wisent, 319–007-CL) containing 10% of heat-inactivated foetal bovine serum (Sigma, #F1051) and 100U/mL of penicillin and streptomycin (Wisent, #450–201-EL) under standard culture conditions (37°C at 5% CO_2_). HiPSCs were cultured and maintained on hESC-qualified Matrigel^®^ (Corning, #08–774–552)-coated plates in Gibco StemFlex medium (ThermoFisher, #A3349401) under standard culture conditions. 12-well plates were coated with Matrigel™ in DMEM/F12 (ThermoFisher, #11330–032) for 1 h at room temperature. For passaging, hiPSC colonies were dissociated in small clumps using ReLeSR™ (Stem Cell technologies, #05872) every 4–7 days. Medium was changed every 2 days. StemPro Accutase (ThermoFisher, #A1110501) was used for hiPSCs dissociation into single cell suspensions.

### 2.3 Reprogramming of fibroblasts lines

One control and one LSFC fibroblast line (7.50E5 cells) were electroporated with 10ug of Addgene episomal vectors (2.5ug of each pCE-hOCT3/4 (#41813), pCE-hSK (#41814), pCE-hUL (#41855) and pCE-mp53DD (#41856)) using the primary mammalian fibroblasts nucleofector kit and U-023 program (Lonza, #VPI-1002). Two days after electroporation, fibroblast medium was replaced by TeSR^TM^-E7^TM^ reprogramming medium (StemCell, #05910). First morphological changes occurred after 10 days in culture and colonies with typical ES cell-like morphology appeared ∼25 days after electroporation. hiPSC colonies were selected via live microscopy (Nikon Eclipse Ti (filter 41018) microscope) using an antibody targeting TRA-1-60 pluripotency marker coupled to DyLight™ 488 fluorophore. Fluorescently TRA-1-60 positive colonies, tightly packed and with well-defined borders were picked from the fibroblast cell layer. At this stage of the reprogramming, each of the selected hiPSC colonies were referred to as clones. Characterization of clones was performed as followed: hiPSC clones were genotyped for LRPPRC*A354V mutation and were tested for reprogramming vector integration by polymerase chain reaction (PCR). mRNA expression of pluripotency markers (*POU5F1, NANOG* and *SOX2*) was assessed by quantitative PCR (qPCR). HiPSC clones were also tested for their ability to differentiate into each of the three germ layers and for chromosome abnormalities and copy number variations using the KaryoStat Karyotyping Service from Invitrogen. Protocols for hiPSCs characterization are detailed in Supplementary Material and methods.

### 2.4 Differentiation of hiPSC into three germ layers

Ectoderm was obtained using the Human Three Germ Layer kit (R&D systems, #SC027) as described in manufacturer’s protocol. Definitive endoderm (DE) was obtained using DE StemDiff media (StemCell technologies, #05110) according to manufacturer’s instructions. To obtain mesodermal cell lineage, hiPSCs at a confluency of 80%–90% were treated with 5 µM CHIR99021 (Sigma, #SML1046) in RPMI-1640 supplemented with B27 minus (−)insulin (ThermoFisher, #A1895601) for 48 h. Medium without CHIR99021 was then added to cells 24 h Three germ layer markers (Otx2, SOX17 and Brachyury) expression were assessed by immunofluorescence (IF) microscopy and by Western Blot (WB). For IF and WB protocols, refer to Supplementary Material and methods.

### 2.5 Differentiation of hiPSCs into hiPSC-HLCs and hiPSC-CMs

HiPSCs were differentiated into hiPSC-HLCs according to Peters *et al.* (Peters et al., 2016) except for definitive endoderm (DE) differentiation which was obtained using DE StemDiff media according to manufacturer’s instructions. For detailed protocol, refer to Figure 3A.

Differentiation of hiPSCs into hiPSC-CMs cells as well as hiPSC-CMs passaging were performed as described by Sharma *et al.* without major modification (Sharma et al., 2015). For detailed protocol, refer to Figure 4A.

### 2.6 ATP quantification

ATP quantification was assessed according to manufacturer’s instructions (Perkin Elmer, #6016943) except that 10uM of Hoeschst 33342 viability stain (ThermoFisher, #H3570) was added 1 h prior cell lysis to control for cell number and viability. Luminescence results were reported to an ATP standard curve and normalized to fluorescence of Hoechst (Ex/Em; 355/460 nm).

### 2.7 Statistical analysis

Each experiment was realized in two CTRL and two LSFC hiPSC clones. Results are presented as the mean of two clones and error bars are the standard deviation (SD), unless specified otherwise in figure legends. Technical replicates are indicated in figure legends.

## 3 Results

With the objective of establishing an experimental model to enable the investigation of tissue-specific differences observed in LSFC patients, we set out to generate hiPSC lines from fibroblast lines established from a patient with LSFC as well as from a sex and age-matched control and then differentiate these into the three germ layers, as well as into hepatic and cardiac lineages (Supplementary Figure S1).

### 3.1 Generation of control and homozygous LRPPRC*354V hiPSC lines

**FIGURE 1:**
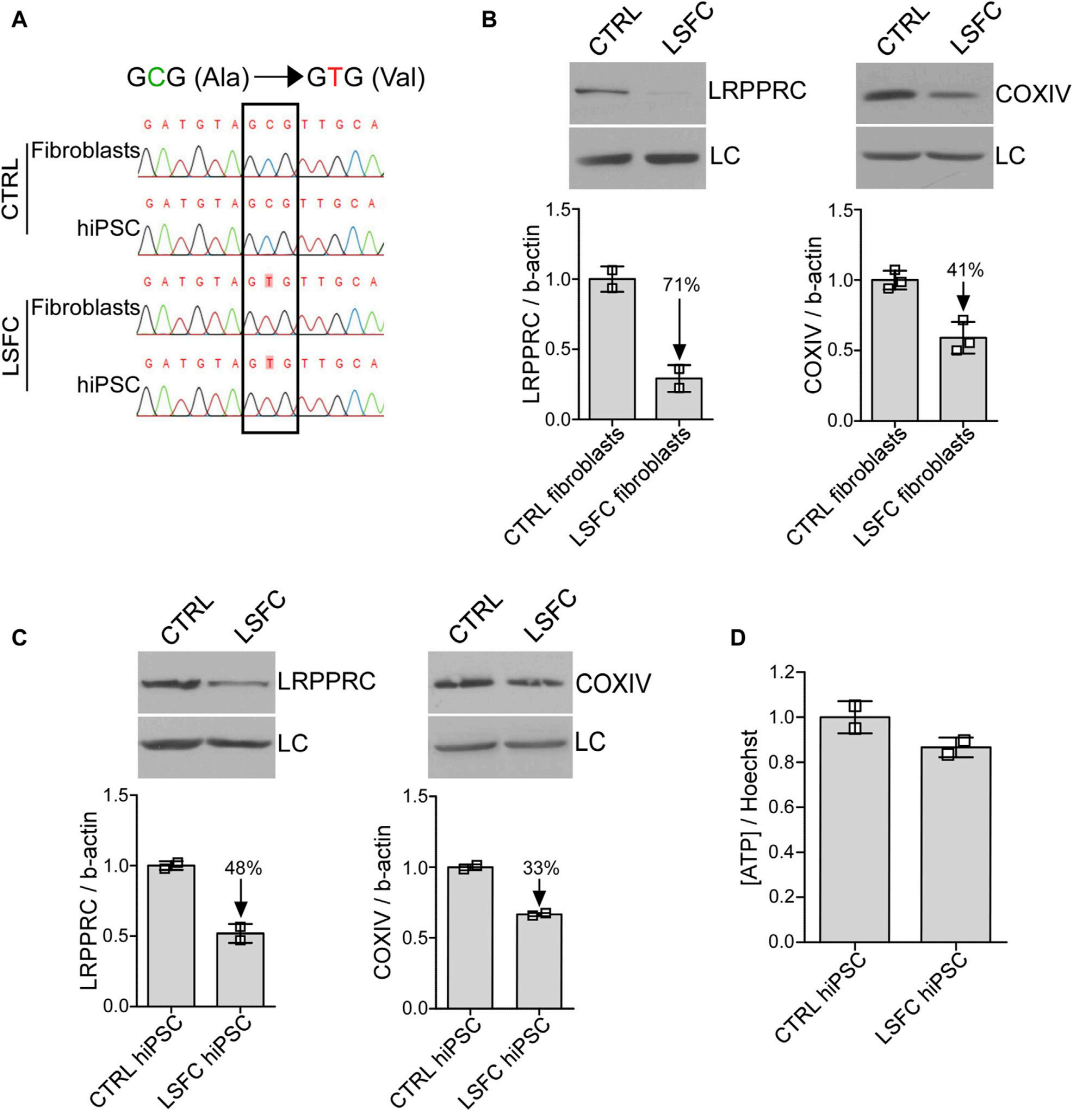
Impact of LRPPRC*354V on LRPPRC and COXIV protein expression in LSFC hiPSCs. **(A)** Genotyping of LSFC and CTRL fibroblasts and hiPSCs for homozygous LRPPRC*354V mutation. **(B, C)** LRPPRC and COXIV average protein expression normalized to β-actin (LC) in CTRL and LSFC fibroblasts (two to three tech. rep. x one cell line) and hiPSCs (one to two tech. rep. x two clones). **(D)** ATP levels in CTRL and LSFC hiPSCs normalized to Hoechst 33342 viability stain (2 clones).

As a first step, one control and one LSFC immortalized fibroblast lines were reprogrammed into hiPSCs using an established 25-day protocol (Supplementary Figure S2A). HiPSC colonies were identified by live cell imaging of the pluripotency marker TRA-1-60 (Supplementary Figure S2B). TRA-1-60 positive colonies, tightly packed and with well defined borders were picked and cultured for an additional five passages. Pluripotency was then confirmed in both hiPSC lines by qPCR for mRNA expression of pluripotency markers *POU5F1*, *NANOG* and *SOX2* (Supplementary Figure S2C). Furthermore, characterization of each hiPSC clones found a normal human karyotype in all (Supplementary Figure S2D). Absence of integration of the reprogramming plasmids was verified by PCR in all selected cell lines (Supplementary Figure S2E). The genotype at the LRPPRC*A354V mutation was validated in fibroblasts and hiPSC lines from the LSFC patient (V/V) and control individual (A/A) (Figure 1A).

### 3.2 LRPPRC*354V causes a decrease in LRPPRC protein levels in fibroblast and hiPSC lines

We next looked at the impact of the LRPPRC*354V mutation on LRPPRC protein levels by WB. As illustrated in Figure 1B, LRPPRC levels were reduced by 71% in LSFC fibroblasts compared to controls. Similarly, the LRPPRC*354V mutation was associated with a 49% decrease of COXIV protein expression in fibroblasts from the LSFC patient as compared to control fibroblasts (Figure 1B). While less pronounced, lower protein levels of LRPPRC (decreased by 48%) and COXIV (decreased by 33%) were also observed in LSFC hiPSCs when compared to the control cells (Figure 1C). Despite lower LRPPRC and COXIV expression, ATP levels in LSFC hiPSCs compared to control hiPSCs were very similar (Figure 1D), although these results should be considered putative given the limitation in the number of individuals studied.

### 3.3 LRPPRC protein levels are reduced in all three embryonic germ layers in LSFC as compared to controls

**FIGURE 2:**
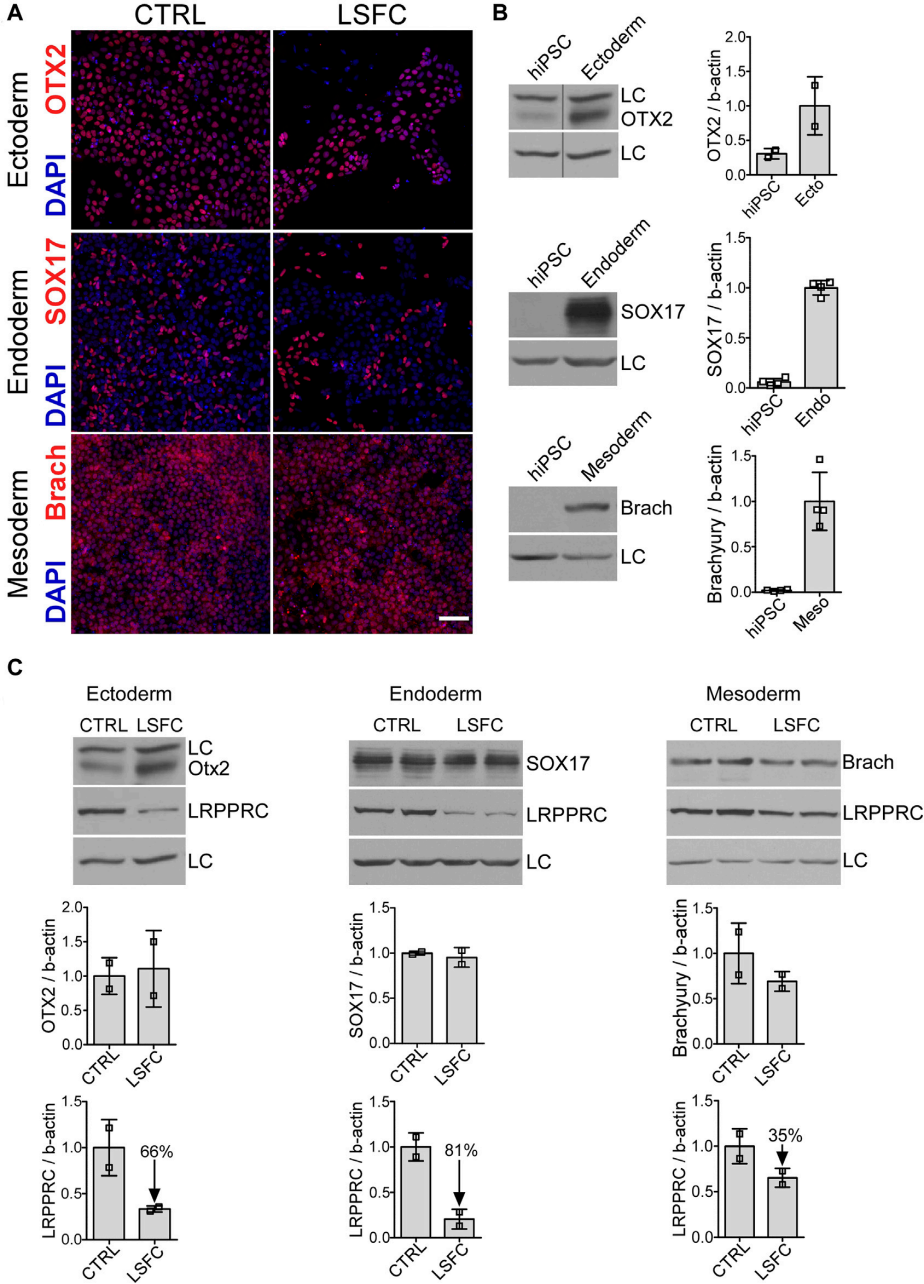
Characterization of cells from the three germ layers derived from LSFC hiPSCs. **(A)** Successful differentiation of LSFC and CTRL hiPSCs into the three germ layers demonstrated by IF staining of OTX2, SOX17 and Brachyury (red) as marker of ectoderm (ecto), definitive endoderm (endo) and mesoderm (meso), respectively. Nuclei (blue) were stained using DAPI (scale bar = 100 μm). **(B)** Gain in the expression of OTX2, SOX17 and Brachyury in the three germ layers derived from CTRL and LSFC hiPSCs demonstrated by WB (2 clones). **(C)** Protein levels of OTX2, SOX17 and Brachyury as well as LRPPRC in ectoderm, definitive endoderm and mesoderm derived from CTRL and LSFC hiPSCs. Bars are the average expression normalized to β-actin of two clones, except for ectoderm where the mean is representative of one clone from two independent differentiations.

Given the biochemical and clinical tissue-specific manifestations in LSFC patients, we were interested in evaluating the effect of the LRPPRC*354V mutation on the steady-state levels of the protein in the three primary germ layers of embryonic development. Control and LSFC hiPSCs were therefore differentiated into ectoderm, definitive endoderm and mesoderm (Supplementary Figure S1). Successful differentiation was achieved for the three germ layers for LSFC and control lines as demonstrated by confocal IF analysis of known markers of the three germ layers: OTX2 (ectoderm), SOX17 (definitive endoderm) and brachyury (mesoderm) (Figure 2A). This was further validated by WB analysis of cell lysates, where prominent increases of these markers between hiPSCs and the different germ layers were observed (Figure 2B). This analysis also revealed that while there was little to no difference in the expression of germ layer markers between control and LSFC cells, there was a marked difference in the level expression of the LRPPRC protein (Figure 2C). Specifically, we observed that LRPPRC expression was reduced by 66% in ectoderm, by 81% in endoderm, and by 35% in mesoderm (Figure 2C) in LSFC cells compared to controls.

### 3.4 HiPSC-based models are consistent with tissue-specificity observed in LSFC

As a next step in developing tissue-specific models of LSFC, we differentiated the hiPSC lines into hiPSC-HLCs and hiPSC-CMs, as it is known that the liver is severely affected in LSFC patients whereas heart functions are essentially unperturbed.

#### 3.4.1 LRPPRC protein is undetectable in LSFC hiPSC-HLCs

**FIGURE 3:**
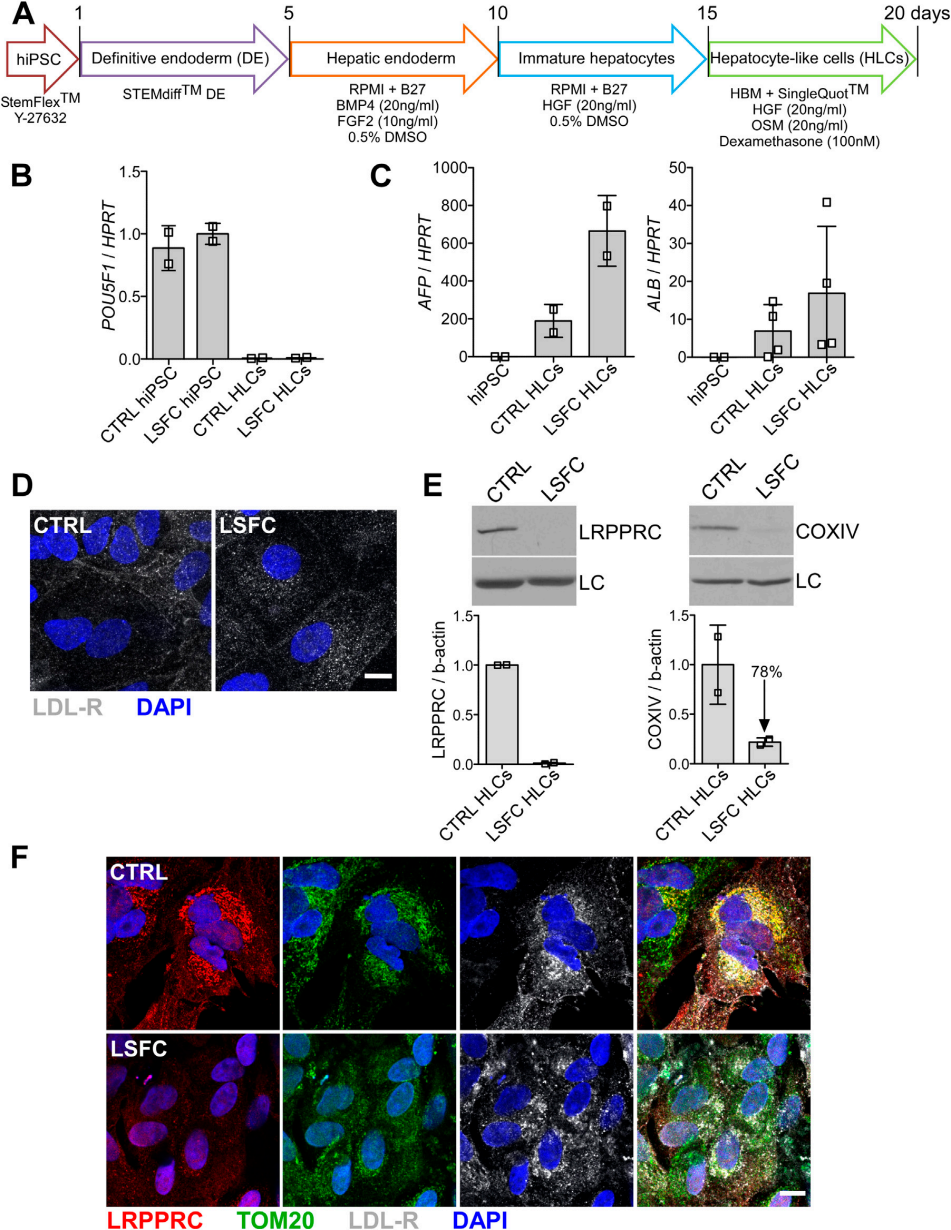
Characterization of hiPSC-HLCs from an LSFC patient. **(A)** Schematic representation of differentiation stages to obtain hiPSC-HLCs. **(B)** Pluripotency marker, (*POU5F1*) and **(C)** hepatic markers (*AFP* and *ALB*) relative mRNA expression normalized to *HPRT* in hiPSC-HLCs. Bars are the average expression of two clones from two independent differentiations. **(D)** Immunofluorescence staining of LDL-R (green) at the cell periphery of hiPSC-HLCs and nuclei (blue) using DAPI (scale = 10 µm). **(E)** LRPPRC and COXIV average protein levels in CTRL and LSFC hiPSC-HLCs of two clones from two independent differentiations. **(F)** IF staining of LRPPRC (red), TOM20 (green) as a marker of the mitochondria, LDL-R (grey) and nuclei (blue) in CTRL and LSFC hiPSC-HLCs (scale bar = 10 μm).

HiPSCs were differentiated into hepatocyte-like cell lines (HLCs) following a 21-day protocol illustrated in Figure 3A. Following this differentiation protocol, we observed that the cells had developed a polygonal shape, consistent with the morphology of hepatocytes. Loss of mRNA expression of pluripotency markers was evaluated in hiPSC-HLCs as well as the presence of hepatic markers. Specifically, we observed that the *POU5F1* and *NANOG* expression that was detected in the hiPSC lines was lost in differentiated hiPSC-HLCs (Figure 3B) and this was associated with an increased expression of the hepatic markers alpha-fetoprotein (*AFP*) and albumin (*ALB*) (Figure 3C). We also confirmed cell surface expression of the LDL receptor in hiPSC-HLCs by IF (Figure 3D). Regarding the impact of the LRPPRC*354V mutation on LRPPRC and COXIV protein expression, we observed via WB analyses revealed that LRPPRC was essentially undetectable and that COXIV was reduced by ∼80% in hiPSC-HLCs derived from LSFC patient compared to control hiPSC-HLCs (Figure 3E). In addition, our IF analyses demonstrated that LRPPRC in control hiPSC-HLCs co-localizes with the mitochondrial marker TOM20 and confirmed that the level of LRPPRC was greatly reduced in hiPSC-HLC cells from the LSFC patient as compared to control (Figure 3F).

#### 3.4.2 LRPPRC protein levels are modestly reduced in LSFC hiPSC-CMs

**FIGURE 4:**
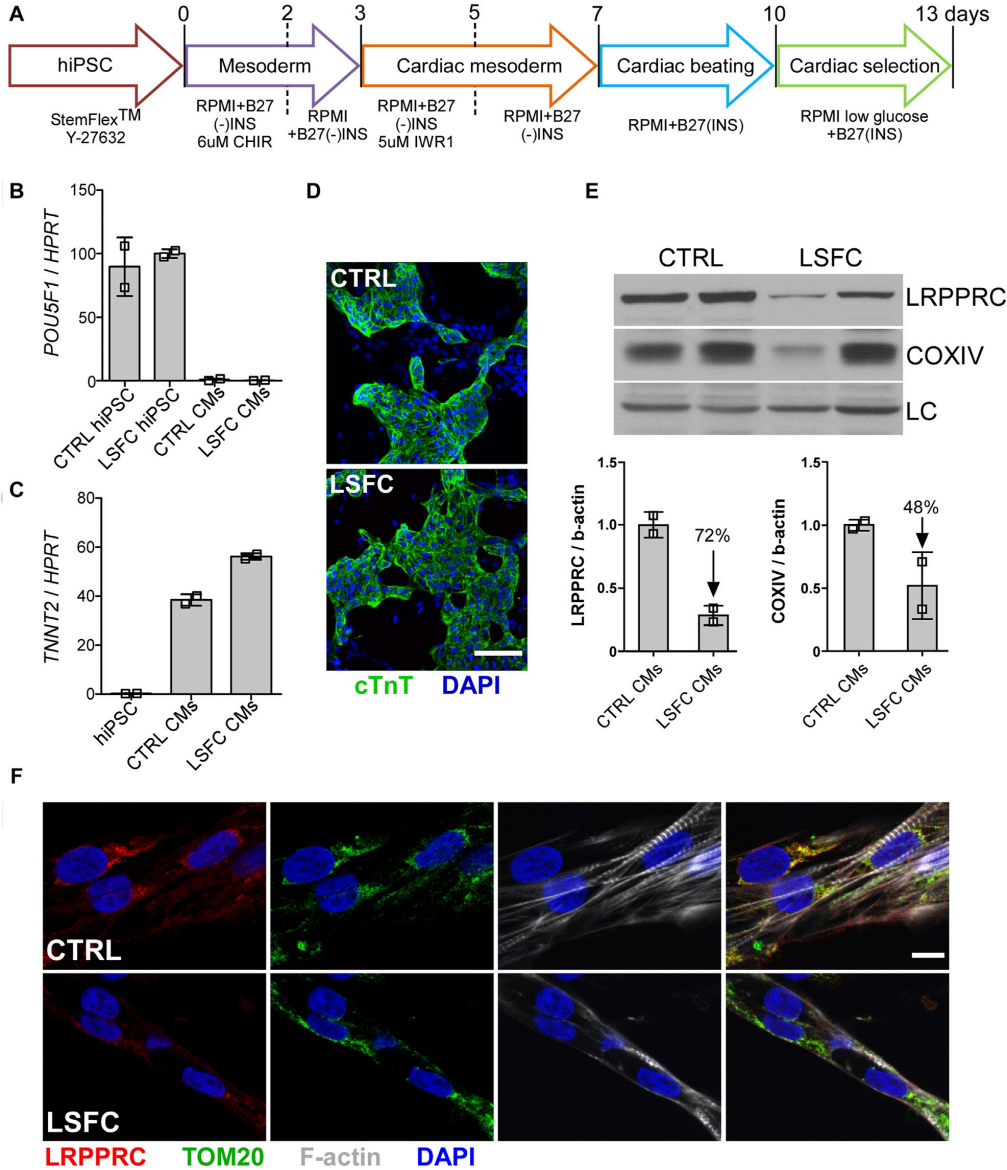
Characterization of hiPSC-CMs from an LSFC patient. **(A)** Schematic representation of differentiation stages to obtain beating hiPSC-CMs. **(B)** *POU5F1* and **(C)** *TNNT2* mRNA relative expression normalized to *HPRT* in hiPSC-CMs. **(D)** IF staining of cTnT (green) and nuclei (blue) using DAPI (scale bar = 100 µm) in CTRL and LSFC hiPSC-CMs. **(E)** LRPPRC and COXIV protein levels in CTRL and LSFC hiPSC-CMs (2 tech. rep. x one to two clones). **(F)** IF staining of LRPPRC (red) and TOM20 (green) in hiPSC-CMs. Phalloidin (grey) was used to stain striated F-actin and DAPI (blue) to stain nuclei (scale bar = 10 μm).

Successful differentiation of control and LSFC hiPSCs to hiPSC-CMs was achieved following a 13-day differentiation protocol with spontaneously beating cells observed in both cell lines at Day 7 (Figure 4A). Moreover, hiPSC-CMs differentiation was further confirmed by the loss of the pluripotency marker *POU5F1* (Figure 4B) and the gain of expression of the cardiac gene *TNNT2* (Figure 4C). Cardiac troponin T (cTnT) was also detected in differentiated cells by confocal IF imaging (Figure 4D). Although there was no observable difference in the expression of these differentiation markers in hiPSC-CMs derived from LSFC or control, our WB analyses detected a 72% reduction of LRPPRC and 48% lower COXIV protein expression in hiPSC-CMs from LSFC patient as compared to control cells (Figure 4E). This was further supported with IF analyses whereby LRPPRC was detectable in LSFC derived hiPSC-CMs, albeit at a lower level than in control hiPSC-CMs (Figure 4F). Co-localization of LRPPRC and TOM20 in control and LSFC hiPSC-CMs was also confirmed by IF analyses (Figure 4F).

### 3.5 Putative association between loss of LRPPRC and altered expression of ER stress markers in hiPSC-HLCs

We investigated the expression of key ER stress markers in our cell lines. We first looked at the transcript levels of key genes involved in ER stress in control and LSFC fibroblasts, hiPSCs, hiPSC-HLCs and hiPSC-CMs. Among all cell types analyzed, our results revealed a trend for increased mRNA levels of *CHOP* in hiPSC-HLCs (Supplementary Figure S3A) as well as a significant increase (60%) of BAX mRNA levels in LSFC hiPSC-HLCs compared to control cells (Supplementary Figure S3B). Transcript level of *HSP5A,* which encodes Bip/Grp78, also increased by ∼25% in LSFC hiPSC-HLCs compared to control cells (Supplementary Figure S4). In contrast, our WB analyses showed that protein expression of BiP/Grp78 was reduced by 35% in LSFC-derived hiPSC-HLCs, while it remained unchanged in the other cells tested (Supplementary Figure S3C). Together, these results show altered expression of key markers of ER stress in hiPSC-HLCs, but not hiPSC-CMs, derived from LSFC patient compared to control cells and not in other cell types tested.

## 4 Discussion

LSFC is a recessive disorder characterized by tissue-specific deficiency in COX and OXPHOS capacity which is associated with a spectrum of clinical manifestations. (Morin et al., 1993; Debray et al., 2011). Since identification of *LRPPRC* as the causal gene for LSFC in 2003, numerous studies have demonstrated its multiple biological roles such as mitochondrial gene translation (Mili and Pinol-Roma, 2003), regulating autophagy (Zou et al., 2013), and tumorigenesis (Tian et al., 2012) and have begun to elucidate its role in the pathogenesis of LSFC. None of the existing models, however, enable the study of the tissue-specific differences in pathology or the tissue-specific differences in LRPPRC expression observed in children affected with LSFC, most of whom are homozygous for the LRPPRC*354V mutation. Interestingly, studies conducted in hiPSC lines from patients with other mitochondrial diseases have proven very valuable for functional studies (Ma et al., 2015; Hattori et al., 2016; Zheng et al., 2016; Galera-Monge et al., 2020). This study was thus undertaken to evaluate if hiPSC-derived cells from LSFC patient could be used as an additional and complementary tool for LSFC research and to specifically assess the tissue-specific impact of LRPPRC*354V. We show here the first time that it is possible to generate hiPSCs from an LSFC patient harboring the LRPPRC*354V homozygous mutation and to differentiate them in various cell lines that can be used to assess the multisystem complexity of this severe metabolic disease.

In this proof-of-principle study, we observed that hiPSCs derived from a patient homozygous for the LRPPRC*354V mutation showed decreased levels of LRPPRC as compared to control hiPSCs homozygous for the ancestral LRPPRC*A354 allele. HiPSCs were also successfully differentiated in hiPSC-CMs and hiPSC-HLCs. Protein expression analyses showed that while LRPPRC is undetectable in LSFC hiPSC-HLCs its expression was partially reduced (by 70%) in LSFC hiPSC-CMs. This differs from the absence of LRPPRC expression as previously observed in total heart tissue from one LSFC patient post-mortem (Sasarman et al., 2015). Regarding the impact of the mutation on the protein levels of COX, they were reduced by 78% in hiPSC-HLCs and by 48% in hiPSC-CMs from LSFC compared to control. Despite changes in LRPPRC and COXIV expression, ATP content as similar in LSFC cells compared to control, at least in hiPSCs cultured in basal conditions. Moreover, preliminary data looking at ATP content in LSFC hiPSC-HLCs compared to control hiPSC-HLCs revealed no major changes in basal condition as well as following 4 h of incubation with 1 mM palmitate, a condition used to mimic metabolic stress. This suggests that altered ATP production is not the primary impact of the mutation in LSFC. Unchanged ATP content has also been previously reported in fibroblasts from LSFC patients despite presence of multiple functional abnormalities in mitochondria, this could be the result of increased glycolytic flux (Burelle et al., 2015; Mukaneza et al., 2019).

As LSFC is a developmental disease, we performed exploratory analyses of the three primary germ layers derived from the hiPSC lines from the LSFC patient and control individual. When comparing these, we observed that the lines from the LSFC patient were noticeably different from the control lines. Specifically, we observed that the mesoderm, from which derives cardiac muscle, seems to be the least affected (35% reduced protein expression) than ectoderm (66% reduced protein expression) and definitive endoderm (81% reduced protein expression), from which derive respectively neural and gastrointestinal tract tissues, including the liver. These results suggest that the impact of LRPPRC*354V could start as early as during embryogenesis and persist beyond. This is consistent with our observations that LRPPRC*354V knock-in mice embryos were not viable past 10 days of gestation, limiting the window for experimental studies to address embryogenesis. Further studies are however needed to deepen our understanding of the impact of LRPPRC*354V during early development. Taken together our analyses performed in hiPSC-derived cell lines suggest that the impact of the LRPPRC*354V mutation on the LRPPRC protein expression varies depending on cell type. This suggest that hiPSC derived cell lines, complementary to the other cell-based models for LSFC as well as tissue-specific LRPPRC conditional knock-out mice, are relevant experimental models to assess the tissue-specificity impact of the mutation.

In our exploratory studies to elucidate the impact of LRPPRC*354V mutation in hiPSC-derived cell lines, we have studied the expression of genes and protein associated with ER stress response or unfolded protein response (UPR). The roles of ER stress in LSFC phenotype is unknown, however, it has been shown in mammalian cell cultures and *C. elegans* that knocking down the expression of LRPPRC (or LRPPRC-like gene mma-1 in C. elegans) triggers transient activation of the mitochondrial UPR (mtUPR). CHOP is the highest inducible gene during ER stress and is one of the first factors involved in mediating the mtUPR, which is an essential mediator of a response to mitochondrial dysfunction (Anderson and Haynes, 2020). Our preliminary data suggest increased mRNA levels for *CHOP* and *BAX* in LSFC hiPSC-HLCs. This increased in *CHOP* transcript level is consistent with what has been reported in whole liver of mouse with hepatocyte-specific inactivation of *Lrpprc* (Cuillerier et al., 2021). We also looked at the expression of BiP, also known as GRP78, a regulator of ER homeostasis (Pfaffenbach and Lee, 2011). We observed that while *HSPA5* transcript levels were increased, BiP protein expression was reduced specifically in hiPSC-HLCs from LSFC patient. The exact impact of this discordance between transcript and protein BiP/GRP78 levels remains to be assessed. However, similarly altered expression of ER stress and mtUPR proteins, increased CHOP and Bcl-2 related proteins and decreased BiP levels, are observed in human hepatic tissue with nonalcoholic fatty liver disease (NAFLD) (Lee et al., 2017). This is of interest considering that mice with hepatocyte-specific deletion of *Lrpprc* as well as LSFC patients present with major lipid changes evocative of NAFLD, including increased triglyceride and long-chain acylcarnitine levels and altered phospholipid content (Ruiz et al., 2019). Further studies are needed to assess the exact impact of these changes in LSFC physiopathology. However, considering that persistent ER stress triggers the basic mechanisms involved in the development or pathology of many diseases including metabolic, neurodegenerative and inflammatory diseases (Chaudhari et al., 2014; Wang and Kaufman, 2014; Zhu et al., 2014), our results reinforce the idea that altered mtUPR in the liver could partially underlie liver impairment in LSFC patients.

In conclusion, we report here for the first time the successful generation of hiPSC-derived cells from LSFC patient. While the current findings need to be validated in a larger number of cell lines derived from independent patients, our study highlights the relevance of hiPSC-derived models for studying the mechanistic and tissue-specific impacts of the LRPPRC*354V mutation, with the hope that this model will also contribute to the future development of therapies for this currently incurable disease.

## Data Availability

The raw data supporting the conclusion of this article will be made available by the authors, without undue reservation.
