# Supplementary material for: Human induced pluripotent stem cells (hiPSCs) derived cells reflect tissue specificity found in patients with Leigh syndrome French Canadian variant (LSFC)
Source: Front Genet. 2024 Apr 19;15:1375467. doi: 10.3389/fgene.2024.1375467 (PMC11066297; doi:10.3389/fgene.2024.1375467)
Supplement: Supplementary file 1 [file Image1.pdf]

Supplementary Material

1 Supplementary Methods

Genotyping of fibroblast and hiPSC lines

Genomic DNA (gDNA) was isolated from the fibroblast and hiPSC lines using the DNeasy Blood & Tissue Kit (Qiagen, #69504). The A354V mutation was amplified by PCR using HotStarTaq plus DNA polymerase (Qiagen, #203605) and specific primer pair listed in table S1. PCR conditions were as follows: initial heat inactivation at 95°C for 5 minutes followed by 35 cycles at 94°C for 30 seconds, 53°C for 30 seconds, 72°C for 60 seconds and final extension at 72°C for 10 minutes. PCR products were validated by Sanger sequencing using 3730xl DNA Analyzer (Applied Biosystems) and DNA sequences were analysed with CLC DNA Workbench software 7 from Qiagen.

Detection of reprogramming plasmid integration by qPCR

To confirm absence of plasmid integration following reprogramming, gDNA of hiPSC at passage 5 was isolated using DNeasy Blood & Tissue Kit (Qiagen, #69504). 60ng of gDNA were amplified by PCR for the detection of pCE-hOCT3/4, pCE-hSK, pCE-hUL and pCE-mp53DD using HotStarTaq plus DNA polymerase (Qiagen, #203605) and specific primers listed in table S1. PCR conditions were as follows: initial heat inactivation at 95°C for 5 minutes followed by 30 cycles at 94°C for 30 seconds, 62°C for 45 seconds, 72°C for 90 seconds and final extension at 72°C for 10 minutes. Plasmid DNA of each reprogramming vectors were used as positive control, parental fibroblasts were used as negative control, water as no temple control (NTC) and *CXCL2* as an internal control gene. 25ul of the PCR reaction were loaded on a 1% agarose gel to visualize plasmid integration.

RNA isolation, complementary DNA synthesis and qPCR

Total RNA from all cell types except hiPSC-CMs was isolated using the RNeasy Plus Mini Kit (Qiagen, #74136), including the optional DNAse treatment (Qiagen, #79254), as described by the manufacturer. For hiPSC-CMs, lysates were treated with 0.1mg Proteinase K (Qiagen, #19131) at 55˚C for 10 minutes prior to RNA isolation with the RNAs with the RNeasy Plus Micro Kit (Qiagen, #74034). All RNA preparations were quantified by optical density measurements with the Take3™ Micro-Volume Plate (BioTek®). 200ng to 1µg of total RNA were reverse transcribed with the High-Capacity cDNA Reverse Transcriptase (ThermoFisher, #4368814) and gene expression quantified using PowerUp™ SYBR™ Green Master Mix (ThermoFisher, #A25776) and QuantStudio™ Real-Time PCR System (ThermoFisher). qPCR primers were pre-designed and purchased from Sigma (KiCqStart® SYBR® Green Primer, #KSPQ12012) and from IDT®. See table S1 for primer sequences. Expression data were normalized to Hypoxanthine Phophoribosyltransferase (*HPRT*) gene.

Immunofluorescence microscopy

Cells were washed twice with phenol red-free RPMI-1640 (Wisent, #350-046-CL) before fixation in 2% PFA (Fisher Scientific, # PI28908)-PBS pH 7.2 solution for 15 minutes at room temperature. Cells were washed three times in PBS pH 7.2 (ThermoFisher, #20012-050) and permeabilized in blocking buffer consisting of PBS pH 7.2, 0.1% triton X-100 (v/v) and 2% (w/v) donkey serum (Sigma, #D9663) for 30 minutes. Cells were immunolabeled with primary antibodies (see table S2) in PBS pH 7.2 containing 0.01% Triton X-100 and 0.1% donkey serum (antibody buffer) overnight at 4˚C. Cells were washed three times in PBS pH 7.2 and incubated with host-specific secondary anti-IgG AlexaFluor® antibodies (1:500, Invitrogen) and DAPI (Sigma, #D9542) for 1 hour at room temperature. AlexaFluor™-555 Phalloidin (1:40, Invitrogen, #A34055) was used for labelling of F-actin. Cells were washed three times and mounted in a solution of 0.4% DABCO (Sigma, # 290734) in glycerol. Images were acquired with a LSM 710 confocal microscope using a 63x/1.4 oil Plan-Apochromat objective (Zeiss, Oberkochen, Germany) or with the INcell Analyzer 6000 microscope (GE Healthcare Life Sciences) equipped with a 20x/0.75 Plan-Apo objective (Nikon). Resulting images were processed using the ZEN 2012 software (Zeiss, Blue edition) or with Image J software (U.S. National Institutes of Health, Marlyland).

Western blotting

For total protein extraction, cells were dissociated with StemPro Accutase and centrifuged at 300xg for 5 minutes. Cell pellets were washed twice with PBS prior lysis in RIPA buffer (25mM Tris-HCl pH 7.6, 150mM NaCl, 0.1% SDS, 1% NP-40 (v/v), 1% sodium deoxycholate (w/v), 2mM Na3VO4 and 2mM NaF) containing protease inhibitors (Roche, #11873580001) for 10 minutes on ice. Lysates were then centrifuged at 16000xg for 10 minutes at 4˚C. Supernatants were collected and protein concentration determined using the Pierce BCA protein assay (Thermo Fisher, #23225). Proteins were prepared in Laemmli sample buffer (Bio-Rad, #1610747) and boiled for 10 min. Samples were separated by SDS-PAGE electrophoreses. Proteins were transferred to a nitrocellulose membrane (Biorad, #162-0112) for Western blotting. Membranes were incubated 30 minutes at room temperature in TBS-T (Tris-buffered saline (TBS)-0.1% (v/v) Tween-20) supplemented with 5% (w/v) low fat milk powder. Membranes were probed with primary antibodies (table S2) for 1h at room temperature in TBS-T containing 5% (w/v) milk powder followed by peroxidase HRP-conjugated antibodies (1:5000) in the same buffer for 1 hour at room temperature. Membranes were incubated with the Western blot Lightning Plus-ECL reagents (Perkin Elmer, #NEL103001EA) according to the manufacturer's instructions. β-actin (ThermoFisher, #MA5-15739) was used as the loading control (LC) and band intensity quantification was performed using ImageJ software.

2 Supplementary Tables and Figures

2.1 Supplementary Tables

Table S 1. Primers

**Gene**		**Sequence (5' - 3')**
**quantitvative PCR primers**		
POU5F1	forward	GAA GGA GAA GCT GGA GCA AA
	reverse	CTT CTG CTT CAG GAG CTT GG
SOX2	forward	AAC CCC AAG ATG CAC AAC TC
	reverse	GCT TAG CCT CGT CGA TGA AC
Nanog	forward	GAT TTG TGG GCC TGA AGA AA
	reverse	ATG GAG GAG GGA AGA GGA GA
AFP	forward	GAT CCC ACT TTT CCA AGT TC
	reverse	TTT GTT CAT GAA TGT CTC CC
ALB	forward	AGC CTA CCA TGA GAA TAA GAG
	reverse	TTG AAG CAC AGA GAA AAG AG
TNNT2	forward	AGA GAG AGT GGA CTT TGA TG
	reverse	TCC TCT TTC TTC CTG TTC TC
HSPA5	forward	AGG AGG AGG ACA AGA AGG
	reverse	GAG TGA AGG CGA CAT AGG
CHOP	forward	TCA CCA TTC GGT CAA TCA GAG C
	reverse	CA CCA TTC GGT CAA TCA GAG C
BAX	forward	TCT GAG CAG ATC ATG AAG AC
	reverse	TCC ATG TTA CTG TCC AGT TC
HPRT	forward	ATA AGC CAG ACT TTG TTG G
	reverse	ATA GGA CTC CAG ATG TTT CC
	forward	TGG CGT CGT GAT TAG TGA TG
	reverse	CAG AGG GCT ACA ATG TGA TGG
**Genotyping primers**		
LRPPRC	forward	GGA CCG TGA TTT ACT GCA
	reverse	CAG TAG TCT GTT AGC TTC TCC
**PCR primers**		
pCE	forward	AAT CTG TGC GGA GCC GAA A
OCT3/4	reverse	CAG GGT GAG CCC CAC AT
SOX2	reverse	GGG CAG CGT GTA CTT ATC CT
LMYC	reverse	TCA TTC TCC GAG TCG CTT GGG
p53DD	reverse	CCG GAA CAT CTC GAA GCG T
CXCL2	forward	GTC ATA GCC ACA CTC AAG AAT G
	reverse	AGG AAC AGC CAC CAA TAA GC

Table S 2. Antibodies

			APPLICATION	
**ANTIGEN**	**Host**	**Company, Cat No.**	**IF**	**WB**
**TRA-1-60**	mouse	ThermoFisher, A25618	1:50	
**LRPPRC**	rabbit	Sasarman *et al.*, 2015	1:250	1:10000
**COXIV**	rabbit	Cell Signaling, 4844		1:3500
**OTX2**	goat	R&D Systems, 967331	1:1000	1:100
**SOX17**	goat	R&D Systems, 967330	1:1000	1:100
**BRACHYURY**	goat	R&D Systems, 967332	1:1000	1:100
**LDL-R**	goat	R&D Systems, AF2148	1:100	
**TOM20**	mouse	Sigma, MABT166	1:500	
**CTNT**	rabbit	Abcam, ab45932	1:200	
**GPR78/BIP**	rabbit	Abcam, ab21685		1:1000
**B-ACTIN**	mouse	ThermoFisher, MA5-15739		1:5000

2.2 Supplementary Figures

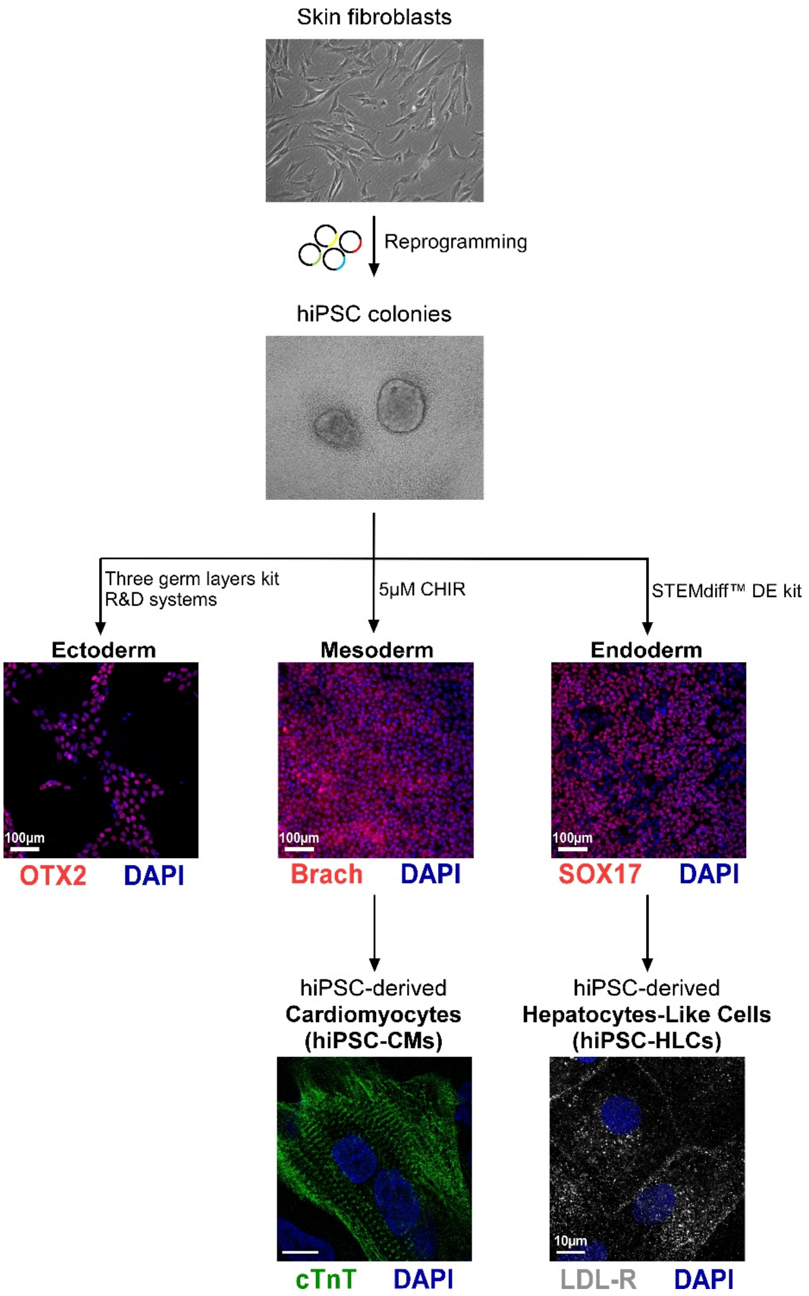

Supplementary Figure 1. Schematic representation of the hiPSC-based approach to study the impact of *LRPPRC354V.** All experiments were conducted in parallel on two hiPSC clones from one control cell line as well as on one cell line from an LSFC patient. Brightfield 4X images were taken using a Nikon Eclipse Ti microscope. OTX2, Brachyury and SOX17 (red) were used as markers of ectoderm, mesoderm, and endoderm, respectively. Cardiac troponin T (green) in hiPSC-CMs and LDL-R (grey) in hiPSC-HLCs were visualized by immunofluorescence and confocal microscopy. DAPI staining (blue) was used to visualize nucleus.

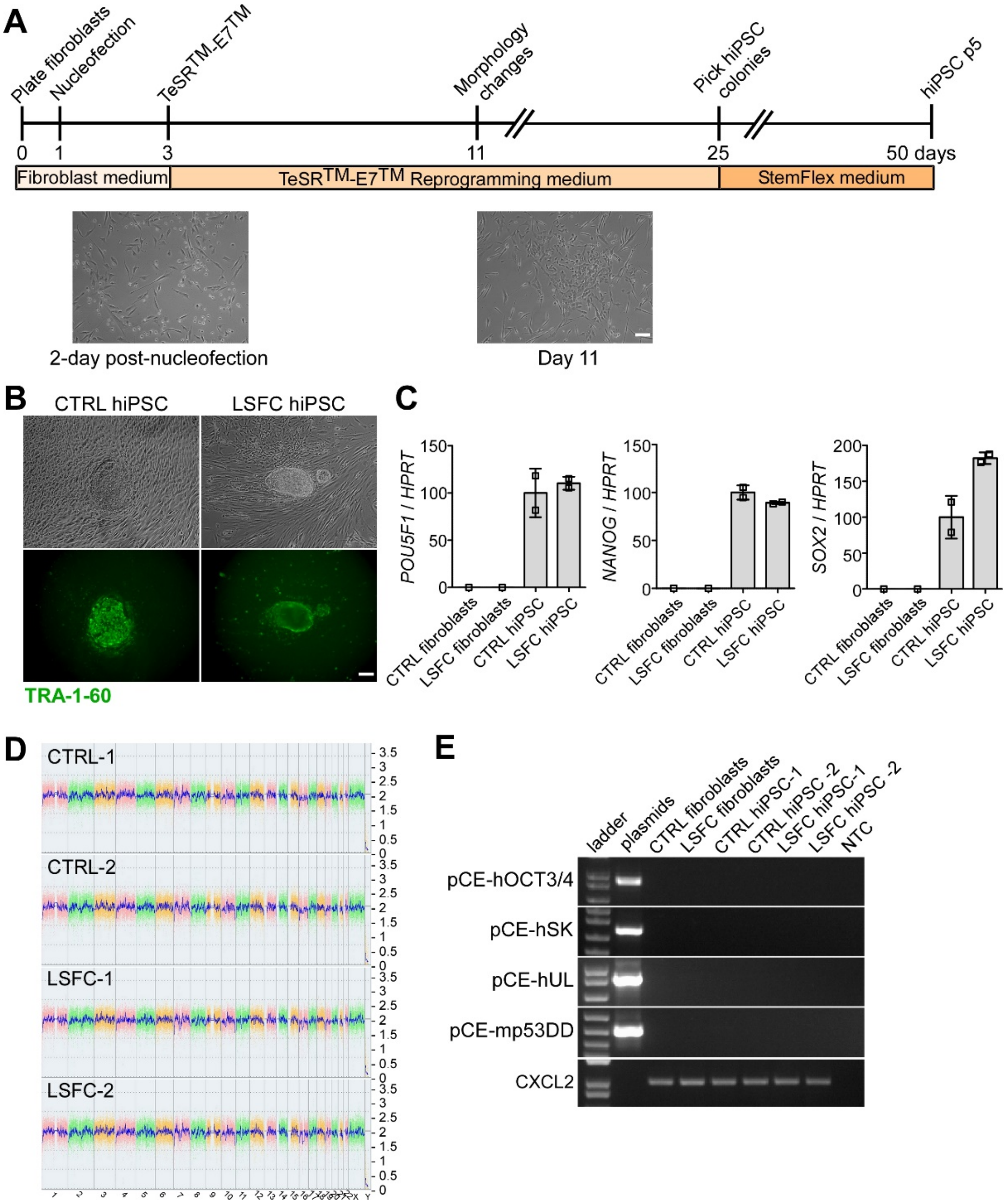

Supplementary Figure 2. Complete characterization of hiPSC derived from fibroblasts from LSFC patient. (A) Schematic representation of reprogramming timeline to obtain hiPSC colonies at passage 5 (p5). (B) Live-cell imaging of TRA-1-60 positive hiPSC clones (green, scale bar = 100µm). (C) *POU5F1*, *SOX2* and *NANOG* relative mRNA expression normalized to *HPRT* in CTRL and LSFC hiPSC clones compared to fibroblasts, as determined by qPCR. Bars are the average gene expression. (D) All hiPSC clones were analyzed by the Karyostat platform (Invitrogen) to confirm the absence of chromosomal abnormalities and copy number variations. (E) Specific primers were used for the detection of reprogramming plasmid integration in all hiPSC clones by PCR. Reprogramming plasmids and fibroblasts were used as a positive and a negative control, respectively and *CXCL2* as an internal control of the PCR amplification. NTC = No Template Control.

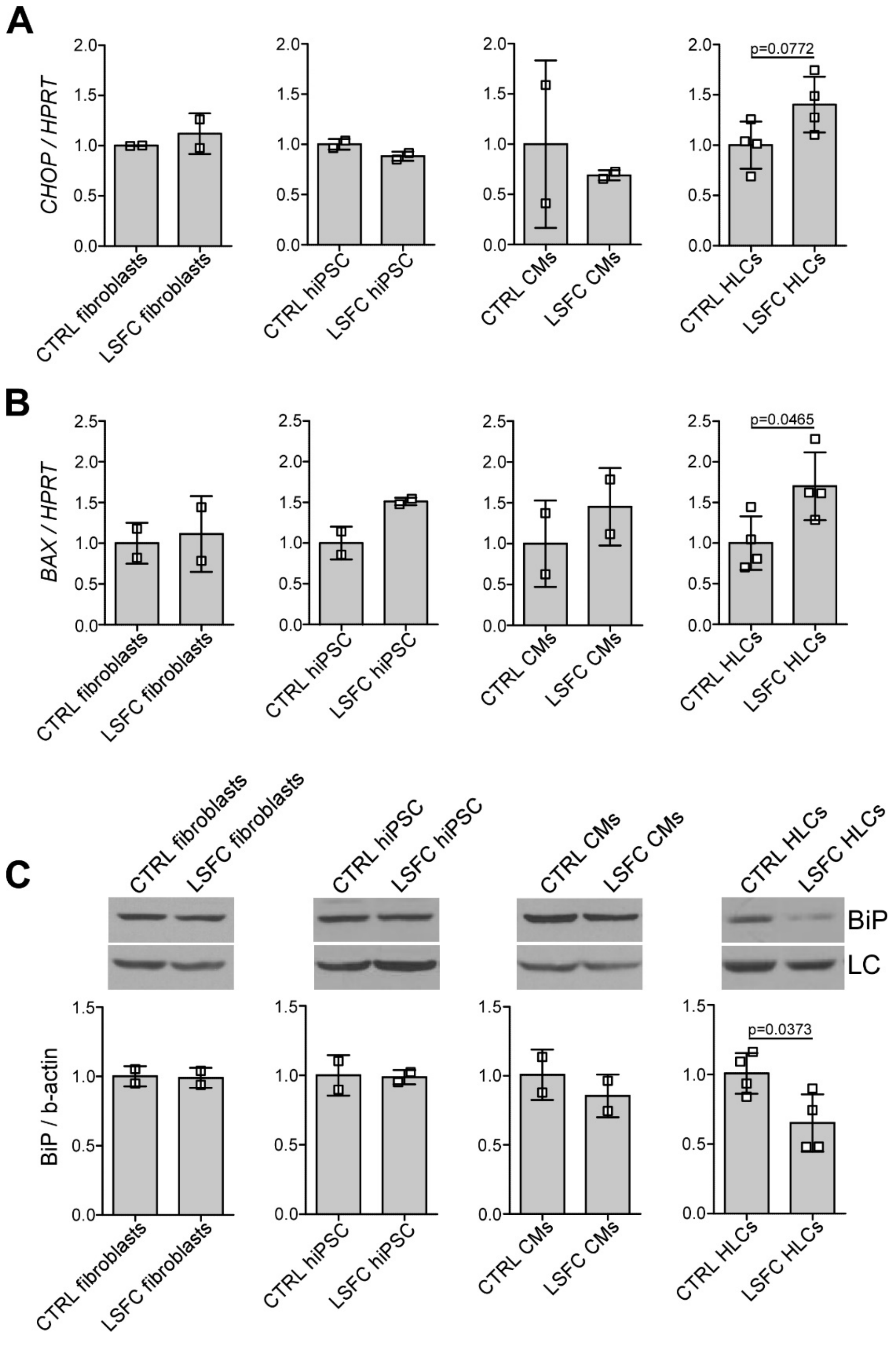

Supplementary Figure 3. Altered ER stress genes and protein expression in cells from LSFC patient. (A-B) *CHOP* and *BAX* mRNA relative expression normalized to *HPRT* in fibroblasts, hiPSC, hiPSC-CMs and hiPSC-HLCs, as determined by qPCR. (C) BiP/GRP78 protein levels as determined by Western Blot. Bars are the average expression normalized to β-actin of two clones except for hiPSC-HLCs where bars are the mean expression of two clones from two independent differentiations. p-values are unpaired *t*-test with Welch's correction.

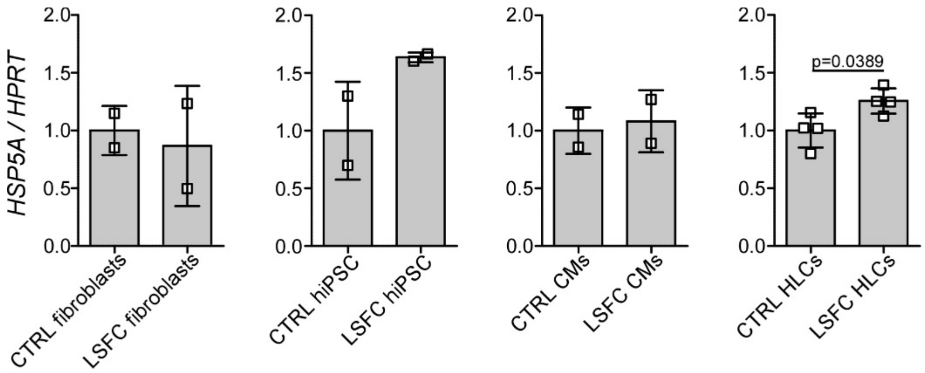

Supplementary Figure 4. *HSPA5* mRNA relative expression normalized to *HPRT* as determined by qPCR. Bars are the average expression of two clones, except for hiPSC-HLCs where bars are the mean expression of two clones from two independent differentiations. p-values are unpaired *t*-test with Welch's correction.
